# Toxoplasma gondii Parasitophorous Vacuole Membrane-Associated Dense Granule Proteins Regulate Maturation of the Cyst Wall

**DOI:** 10.1128/mSphere.00851-19

**Published:** 2020-01-15

**Authors:** Rebekah B. Guevara, Barbara A. Fox, David J. Bzik

**Affiliations:** a Department of Microbiology and Immunology, The Geisel School of Medicine at Dartmouth, Lebanon, New Hampshire, USA; University of Georgia

**Keywords:** *Toxoplasma gondii*, cysts, chronic infection, cyst wall, cyst membrane, parasitophorous vacuole membrane, dense granules, bradyzoite differentiation, cyst development, GRA

## Abstract

After differentiation is triggered, the tachyzoite-stage Toxoplasma gondii parasitophorous vacuole membrane (PVM) has been hypothesized to transition into the cyst membrane that surrounds the cyst wall and encloses bradyzoites. Here, we tracked the localization of two PVM dense granule (GRA) proteins (GRA5 and GRA7) after *in vitro* differentiation of the tachyzoite stage parasitophorous vacuole into the mature cyst. GRA5 and GRA7 were visible at the cyst periphery at 6 h and at all later times after differentiation, suggesting that the PVM remained intact as it transitioned into the cyst membrane. By day 3 postdifferentiation, GRA5 and GRA7 were visible in a continuous pattern at the cyst periphery. In mature 7- and 10-day-old cysts permeabilized with a saponin pulse, GRA5 and GRA7 were localized to the cyst membrane and the cyst wall regions. Cysts at different stages of cyst development exhibited differential susceptibility to saponin permeabilization, and, correspondingly, saponin selectively removed GRA5 from the cyst membrane and cyst wall region in 10-day-old cysts. GRA5 and GRA7 were localized at the cyst membrane and cyst wall region at all times after differentiation of the parasitophorous vacuole, which supports a previous model proposing that the PVM develops into the cyst membrane. In addition, evaluation of Δ*gra3*, Δ*gra5*, Δ*gra7*, Δ*gra8*, and Δ*gra14* mutants revealed that PVM-localized GRAs were crucial to support the normal rate of accumulation of cyst wall proteins at the cyst periphery.

**IMPORTANCE** Toxoplasma gondii establishes chronic infection in humans by forming thick-walled cysts that persist in the brain. Once host immunity wanes, cysts reactivate to cause severe, and often lethal, toxoplasmic encephalitis. There is no available therapy to eliminate cysts or to prevent their reactivation. Furthermore, how the cyst membrane and cyst wall structures develop is poorly understood. Here, we visualized and tracked the localization of *Toxoplasma* parasitophorous vacuole membrane (PVM) dense granules (GRA) proteins during cyst development *in vitro.* PVM-localized GRA5 and GRA7 were found at the cyst membrane and cyst wall region throughout cyst development, suggesting that the PVM remains intact and develops into the cyst membrane. In addition, our results show that genetic deletion of PVM GRAs reduced the rate of accumulation of cyst wall cargo at the cyst periphery and suggest that PVM-localized GRAs mediate the development and maturation of the cyst wall and cyst membrane.

## Abstract (precis)

Toxoplasma gondii establishes chronic infection in humans by forming thick-walled cysts that persist in the brain. Once host immunity wanes, cysts reactivate to cause severe, and often lethal, toxoplasmic encephalitis. There is no available therapy to eliminate cysts or to prevent their reactivation. Furthermore, how the cyst membrane and cyst wall structures develop is poorly understood. Here, we visualized and tracked the localization of *Toxoplasma* parasitophorous vacuole membrane (PVM) dense granules (GRA) proteins during cyst development *in vitro.* PVM-localized GRA5 and GRA7 were found at the cyst membrane and cyst wall region throughout cyst development, suggesting that the PVM remains intact and develops into the cyst membrane. In addition, our results show that genetic deletion of PVM GRAs reduced the rate of accumulation of cyst wall cargo at the cyst periphery and suggest that PVM-localized GRAs mediate the development and maturation of the cyst wall and cyst membrane.

## INTRODUCTION

A third of the global human population is persistently infected with Toxoplasma gondii (1). *Toxoplasma* infection is acquired by ingestion of oocysts in water or on unwashed food, or by ingestion of tissue cysts in undercooked meat (2). While immunocompetent humans commonly control the infection, *Toxoplasma* can cause severe inflammation of the retina leading to ocular toxoplasmosis (3), and during immune suppression, cysts can reactivate in the brain causing toxoplasmic encephalitis (4, 5). The biology underlying the development of tissue cysts remains poorly understood, and current therapies do not target the cyst stage.

Tachyzoite-stage parasites actively penetrate host cells through self-driven motility, and during invagination, the parasite hijacks lipids present in the host cell plasma membrane (6) to establish an intracellular parasitophorous vacuole (PV) (7). During the process of invasion, the tachyzoite also injects bulb contents of the rhoptry organelle into the host cell cytosol (8) to form rhoptry protein (ROP)- and lipid-containing evacuoles that fuse with the nascent parasitophorous vacuole membrane (PVM) shortly after its formation (9). Within a few minutes of PV formation, the contents of the dense granules and the PVM dense granule (GRA) proteins are massively secreted into the lumen of the PV (10). These GRA proteins, together with lipids, are used to establish a nanotubular membranous intravacuolar network (IVN) in the lumen of the PV (11) that connects parasites to one another and to the PVM (12). The secreted GRA proteins can traffic to the PV space, IVN, and PVM, and a subset of GRAs are translocated past the PVM and traffic to the host cytosol or host cell nucleus (13, 14).

The PVM is a dynamic interface that supports parasite replication and survival. ROP17 (15), as well as MYR1, MYR2, and MYR3, are part of a translocon system in the PVM (16–18) that mediates the translocation of GRA16 (19, 20), GRA18 (21), GRA24 (20, 22), GRA28 (23), TgIST (24), HCE1 (25), and TEEGR (26) across the PVM. While the PVM translocation system is crucial for host cell manipulation and parasite survival (27), recent evidence suggests that after stage differentiation into bradyzoites, GRA proteins do not translocate past the cyst wall and cyst membrane, indicating either that the cyst wall is a barrier to GRA translocation or that the cyst membrane lacks a functional translocon (28). Other PVM-localized proteins, such as GRA17 and GRA23, function to facilitate the movement of small molecules or nutrients across the PVM (29, 30). A similar transport mechanism also appears to be active in the cyst stage and is mediated by a GRA17 permeability pore in the cyst membrane (31).

PVM-localized ROP5, ROP17, and ROP18 work in concert to inactivate host immunity-related GTPases (IRGs), which are part of an interferon gamma (IFN-γ)-induced mechanism that targets and eliminates foreign intracellular microbes (32–34). ROP5 prevents IRG oligomerization (35), whereas ROP17/ROP18 phosphorylate IRGs and thus disrupts their ability to attack the PV and eliminate parasites (32, 36, 37). GRA12 also resists host IFN-γ (38). IFN-γ upregulates host guanylate binding proteins (GBPs) (39–41), autophagy proteins (42, 43), and ubiquitin-associated mechanisms that target the PVM to restrict parasite replication in the PV and control infection (44–47). While macrophages harbor the most efficient IFN-γ-activated innate cell autonomous mechanisms that restrict tachyzoite replication in the PV, cysts within the central nervous system develop primarily inside neurons (48). Currently, it is unknown whether neurons can eliminate PVs or cysts via innate cell autonomous immunity or whether the cyst membrane contains parasite proteins that resist clearance mechanisms.

The hallmark of the cyst structure is a thick-walled structure, the cyst wall, that develops underneath the cyst membrane to provide a protective barrier enclosing the bradyzoites (49, 50). The major cyst wall glycoprotein, CST1, contains a mucin domain that is heavily decorated with *N*-acetylgalactosamine moieties that selectively bind to Dolichos biflorus agglutinin (DBA) stain (49, 51, 52). A proteomic study of purified cyst wall and cyst membrane fragments revealed the presence of many dense granule proteins in these structures (53). GRA proteins associated with the IVN (GRA2, GRA4, GRA9, and GRA12) and GRA proteins associated with the PVM (GRA3, GRA5, GRA7, GRA8, and GRA14 [10]) were identified (53). Recently, IVN GRAs were localized in the cyst wall and cyst matrix during cyst development and in mature cysts (54). Deletion of PVM- and IVN-associated GRA proteins significantly reduced cyst burdens in mice without affecting the ability of these GRA mutants to differentiate into bradyzoites and *in vitro* cysts (38, 55). In addition, the IVN GRAs (GRA2, GRA4, GRA6, GRA9, and GRA12) were found to be important for the development and maturation of the cyst matrix and the cyst wall structures (54). Currently, the fate and role of PVM-localized GRA proteins during cyst development is unknown.

The PVM is hypothesized to develop into the cyst membrane (56). A low-CO_2_ and high-pH *in vitro* tachyzoite-to-bradyzoite differentiation method (57, 58) produces mature orally infectious cysts (59) that possess the characteristic cyst structures of *in vivo* cysts isolated from brains of infected mice (60). Using this *in vitro* differentiation method, we investigated PVM GRAs (i) to track the fate of the PVM and its relationship to the development of the cyst membrane and (ii) to test whether PVM GRA proteins influence the development of the cyst wall. Our findings reveal that deletion of PVM GRAs also significantly reduced the rate of accumulation of cyst wall proteins at the cyst periphery relative to that in the cyst interior, suggesting an important role for these PVM GRA proteins in the development of the cyst wall. In addition, our results support the current model proposing that the tachyzoite-stage PVM transitions into the cyst membrane that surrounds the cyst wall.

## RESULTS

### DBA staining intensity at the cyst periphery relative to that in the cyst interior is decreased in mutant strains that lack expression of PVM-associated GRA proteins.

**FIG 1:**
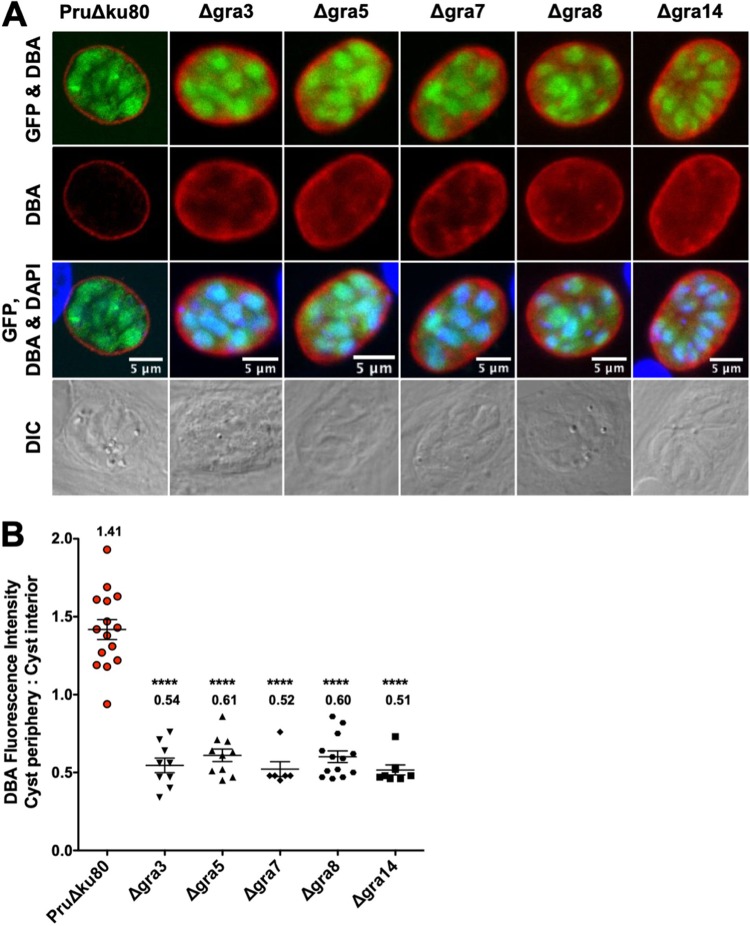
DBA staining intensity at the cyst periphery relative to that in the cyst interior is decreased in mutant strains that lack expression of PVM-associated GRA proteins. (A to B) *In vitro* cysts derived from different GRA deletion strains or the parental PruΔ*ku80* strain were differentiated for 3 days. (A) Cysts were located using differential interference contrast (DIC) microscopy and imaged by confocal microscopy. The presence of bradyzoites inside cysts was verified by locating parasite nuclei with 4′,6-diamidino-2-phenylindole (DAPI) staining and verifying that each parasite nucleus was surrounded by expression of cytosolic green fluorescent protein (GFP) (GFP^+^ bradyzoites). Cysts were stained with Dolichos biflorus agglutinin (DBA). Panels show GFP and DBA; DBA; GFP, DBA, and DAPI; and DIC. Bar, 5 μm. (B) Cysts from each strain were analyzed to determine the ratio of DBA staining intensity at the cyst periphery relative to that in the cyst interior. Data were plotted as the average ratio mean of fluorescence intensity at the cyst periphery to the cyst interior ± standard error of the mean (SEM) for each strain. Δgra3 (*n *= 9), Δgra5 (*n *= 10), Δgra7 (*n *= 6), Δgra8 (*n *= 13), Δgra14 (*n *= 8), and PruΔku80 (*n *= 15). *P* values were calculated with a Student’s *t* test; ****, *P* < 0.0001.

GRA deletion mutants that do not express PVM-associated GRA3, GRA5, GRA7, GRA8, or GRA14 were previously developed in the low-virulence cyst-competent type II PruΔ*ku80* genetic background (38). Recently, we found that the deletion of IVN-associated GRA proteins caused a defect in the development of the cyst wall (38, 54). To determine whether a similar cyst wall defect is observed in mutants that lack expression of PVM GRAs, we measured Dolichos biflorus agglutinin (DBA) fluorescence intensity at the cyst peripheries of Δ*gra3*, Δ*gra5*, Δ*gra7*, Δ*gra8*, and Δ*gra14* cysts (57, 58, 61, 62) (Fig. 1A). DBA selectively stains the major cyst wall protein CST1 (49, 51). DBA fluorescence intensity was quantitatively measured at the cyst periphery, which reflects CST1 cargo delivered to the cyst periphery, and was compared to that in the cyst interior, which reflects CST1 cargo not yet delivered to the cyst wall/membrane. Similar to IVN GRA deletion mutants Δ*gra12* (38) and Δ*gra2*, Δ*gra4*, Δ*gra6*, Δ*gra9* (54), the Δ*gra3*, Δ*gra5*, Δ*gra7*, Δ*gra8*, and Δ*gra14* mutants exhibited a significant decrease in the DBA fluorescence intensity ratio (cyst periphery/cyst interior) (Fig. 1B).

### Localization of PVM-associated GRA5 and GRA7 early after differentiation of the tachyzoite-stage PV.

The cyst membrane is hypothesized to originate from the PVM (56). GRA5 localizes to the tachyzoite stage PVM and to the cyst membrane (63–67). In contrast, GRA7 associates with both the PVM and the IVN membranes in the tachyzoite-stage PV (65, 68). In mature brain cysts, GRA7 localized to membrane tubule components of the intracyst network (ICN) in the cyst matrix, a structure that resembles the IVN, as well as to convoluted membranes that were present in the cyst wall (56). We reasoned that if the cyst membrane originates from the PVM, then soon after differentiation, GRA5 and GRA7 would also localize to the cyst membrane and cyst wall compartments in the developing cyst.

We analyzed the localization of PVM-associated GRAs within the developing cyst 6 h after differentiation of the tachyzoite stage PV. Green fluorescent protein-positive (GFP^+^) bradyzoites and cyst wall proteins stained by DBA were visible within the developing cyst 6 h after differentiation (Fig. S1A-B), showing that alkaline switch differentiation rapidly upregulated expression of bradyzoite-stage genes such as LDH2 and CST1. In contrast, expression of GFP^+^ tachyzoites or DBA cyst wall proteins was not detected in the tachyzoite-stage parasitophorous vacuole (Fig. S1C). These newly differentiated cysts inside host cells were fixed in 4% paraformaldehyde, and cyst membranes were permeabilized using a pulse of saponin or using continuous saponin or Triton X-100 (Triton) detergent treatment. While DBA staining of the 6-h-old cyst was observed with both detergents, cysts were more intensely stained with DBA after permeabilization with Triton (Fig. S1A-B). In addition, consistent with the preservation of the PVM after differentiation is triggered, GRA5 (Fig. S1A) and GRA7 (Fig. S1B) were observed as bright puncta toward the cyst periphery.

10.1128/mSphere.00851-19.1 FIG S1 Parasitophorous vacuole membrane (PVM)-associated GRAs are localized within the developing cyst in 6-hour-old cysts. (A to B) Infected human foreskin fibroblasts (HFFs) on coverslips were treated under bradyzoite-inducing conditions for 6 hours to differentiate *in vitro* cysts. Cysts were located using differential interference contrast (DIC) microscopy and imaged by confocal microscopy. The presence of bradyzoites inside cysts was verified by locating parasite nuclei with 4′,6-diamidino-2-phenylindole (DAPI) staining and verifying that each parasite nucleus was surrounded by expression of cytosolic green fluorescent protein (GFP) (GFP^+^ bradyzoites). Cysts fixed in 4% paraformaldehyde were permeabilized with either a short pulse (P) of saponin or with continuous (C) exposure to Triton. Cysts were stained with DBA and (A) α-GRA5 or (B) α-GRA7 antibody. Panels show GFP and DAPI; GFP and DBA; DBA; α-GRA; DBA and α-GRA; and DIC. Number (*n*) of cysts analyzed, 9 to 18. Bar, 5 μm. (C) Infected HFFs on coverslips were infected with tachyzoites at a multiplicity of infection (MOI) of 0.5, permeabilized with 0.01% saponin, and stained with DBA. Vacuoles were located using DIC microscopy and imaged by confocal microscopy. DAPI-stained host and parasite nuclei. Panels show DAPI; DAPI and GFP; GFP; DBA; GFP and DBA; and DIC. Number (*n*) of vacuoles analyzed, 6. Bar, 5 μm. Download FIG S1, TIF file, 0.3 MB. Copyright © 2020 Guevara et al. 2020 Guevara et al. This content is distributed under the terms of the Creative Commons Attribution 4.0 International license.

### Localization of GRA5 and GRA7 in immature cysts.

**FIG 2:**
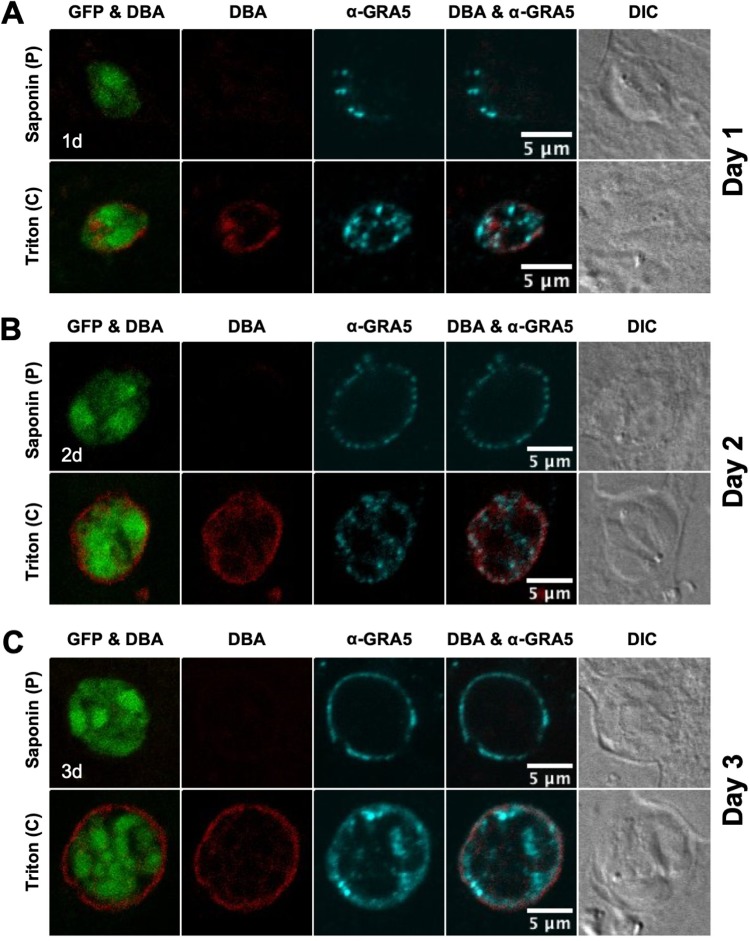
GRA5 localizes to the cyst periphery in immature cysts. (A to C) Infected HFFs on coverslips were treated under bradyzoite-inducing conditions to differentiate *in vitro* cysts for (A) 1 day, (B) 2 days, or (C) 3 days. Cysts were located using DIC microscopy and imaged by confocal microscopy. The presence of bradyzoites inside cysts was verified by locating parasite nuclei with DAPI staining (not shown) and verifying that each parasite nucleus was surrounded by expression of cytosolic GFP (GFP^+^ bradyzoites). Cysts fixed in 4% paraformaldehyde were permeabilized with either a short pulse (P) of saponin or with continuous (C) exposure to Triton. Cysts were stained with DBA and α-GRA5 antibody. Panels show GFP and DBA, DBA, α-GRA5, DBA and α-GRA5, and DIC. Number (*n*) of cysts analyzed, 15 to 32. Bar, 5 μm.

**FIG 3:**
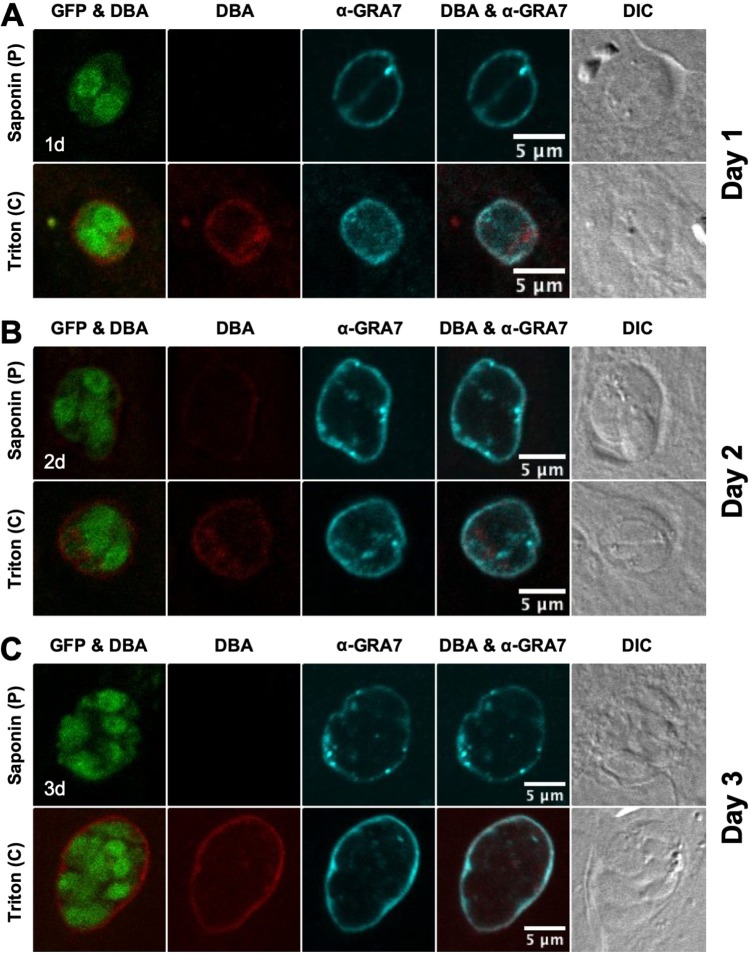
GRA7 localizes to the cyst periphery in immature cysts. (A to C) Infected HFFs on coverslips were treated under bradyzoite-inducing conditions to differentiate *in vitro* cysts for (A) 1 day, (B) 2 days, or (C) 3 days. Cysts were located using DIC microscopy and imaged by confocal microscopy. The presence of bradyzoites inside cysts was verified by locating parasite nuclei with DAPI staining (not shown) and verifying that each parasite nucleus was surrounded by expression of cytosolic GFP (GFP^+^ bradyzoites). Cysts fixed in 4% paraformaldehyde were permeabilized with either a short pulse (P) of saponin, or with continuous (C) exposure to Triton. Cysts were stained with DBA and α-GRA7 antibody. Panels show GFP and DBA, DBA, α-GRA7, DBA and α-GRA7, and DIC. Number (*n*) of cysts analyzed, 9 to 33. Bar, 5 μm.

Immature cysts were differentiated for 1, 2, or 3 days, and the localization of GRA5 (Fig. 2) and GRA7 (Fig. 3) was determined. GFP^+^ bradyzoites were visible within a DBA-stained cyst that was permeabilized with Triton. In contrast, a pulse with saponin did not permeabilize the cyst membrane of 1-, 2-, and 3-day-old cysts to visibly DBA stain the developing cyst wall (Fig. 2 and 3). However, continuous saponin permeabilization was sufficient to reveal DBA staining of the 2- and 3-day-old cyst wall with DBA (Fig. S2). Thus, compared to 6-h-old cysts (Fig. S1A-B), saponin pulse permeabilization had a reduced ability to establish detectable DBA staining of the cyst wall in 1-, 2-, and 3-day-old cysts (Fig. 2 and 3). These results revealed that the cyst wall develops underneath the cyst membrane, and suggests that the lipid composition of the 1- to 3-day-old cyst membrane is more resistant to permeabilization with saponin pulse treatment than that of cysts differentiated for 6 h.

10.1128/mSphere.00851-19.2 FIG S2 GRA5 is localized at the cyst periphery after saponin exposure of 2- and 3-day-old cysts. Infected HFFs on coverslips were treated under bradyzoite-inducing conditions for 2 or 3 days to differentiate *in vitro* cysts. Cysts were located using DIC microscopy and imaged by confocal microscopy. The presence of bradyzoites inside cysts was verified by locating parasite nuclei with DAPI staining (shown in first panel) and verifying that each parasite nucleus was surrounded by expression of cytosolic GFP (GFP^+^ bradyzoites). Cysts fixed in 4% paraformaldehyde were permeabilized with continuous (C) exposure to saponin. Cysts were stained with DBA and α-GRA5. Panels show GFP and DAPI; GFP and DBA; DBA; α-GRA5; DBA and α-GRA5; and DIC. The percent occurrence is shown for GRA5 at day 2 (*n *= 35; 35/35 GRA5 at the cyst periphery) and at day 3 (*n *= 32; 30/32 GRA5 at the cyst periphery). Bar, 5 μm. Download FIG S2, TIF file, 0.1 MB. Copyright © 2020 Guevara et al. 2020 Guevara et al. This content is distributed under the terms of the Creative Commons Attribution 4.0 International license.

GRA5 was localized to the cyst periphery in 1- (Fig. 2A), 2- (Fig. 2B), and 3-day-old (Fig. 2C) cysts. In contrast to a saponin pulse (Fig. 2) or continuous saponin treatment (Fig. S2) that localized GRA5 only at the cyst periphery, GRA5 puncta were also observed within the cyst matrix after cysts were permeabilized with Triton (Fig. 2). GRA7 was also localized at the cyst periphery of 1- (Fig. 3A), 2- (Fig. 3B), and 3-day-old (Fig. 3C) cysts. However, in comparison to GRA5 staining patterns, GRA7 puncta were rarely observed inside the cyst after permeabilization with Triton. Correspondingly, in comparison to saponin treatment, GRA5 peripheral staining was reduced after Triton treatment whereas GRA7 peripheral staining was not reduced (Fig. 2 and 3), revealing that GRA5 associated with the cyst membrane in a different manner than did GRA7. As previously reported using these permeabilization methods (54), staining of dense granules inside the GFP^+^ region that defines bradyzoites was not readily apparent again, indicating that this compartment was not permeabilized (Fig. 2 and 3).

### Localization of GRA5 and GRA7 in mature 7-day-old cysts.

**FIG 4:**
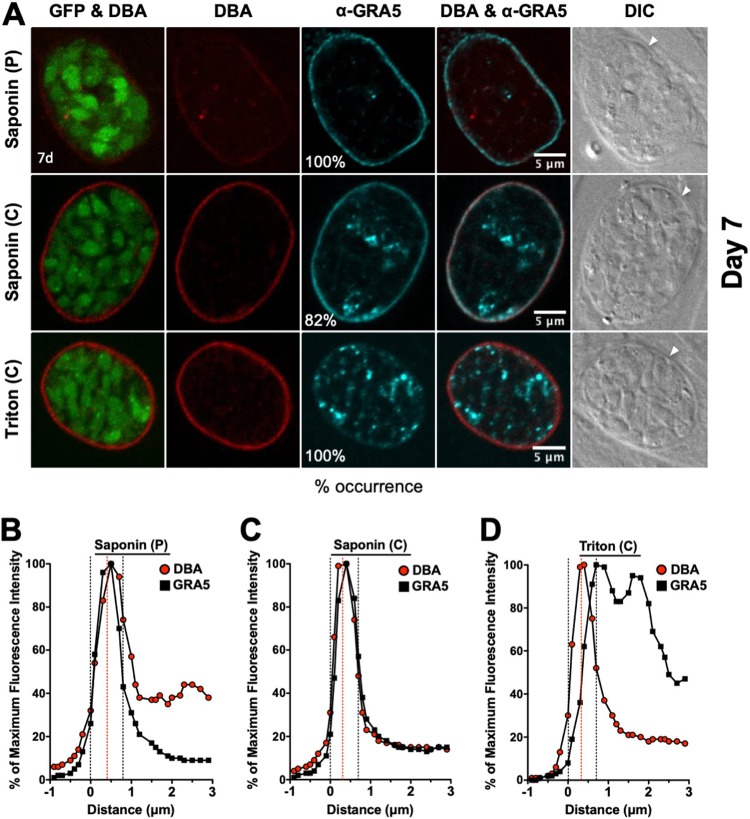
Localization of GRA5 in 7-day-old mature cysts. (A) Infected HFFs on coverslips were treated under bradyzoite-inducing conditions for 7 days to differentiate mature *in vitro* cysts. Cysts were located using DIC microscopy and imaged by confocal microscopy. The presence of bradyzoites inside cysts was verified by locating parasite nuclei with DAPI staining (not shown) and verifying that each parasite nucleus was surrounded by expression of cytosolic GFP (GFP^+^ bradyzoites). Cysts fixed in 4% paraformaldehyde were permeabilized with either a short pulse (P) of saponin, with continuous (C) exposure to saponin, or with continuous (C) exposure to Triton. Cysts were stained with DBA and α-GRA5 antibody. Panels show GFP and DBA, DBA, α-GRA5, DBA and α-GRA5, and DIC (cyst wall indicated by white arrow). The percent occurrence is shown for GRA5 with saponin (P) (*n *= 11; 11/11 GRA5 at the cyst membrane/wall), saponin (C) (*n *= 22; 18/22 GRA5 at the cyst membrane/wall), and Triton (*n *= 9; 9/9 GRA5 not at the cyst membrane/wall). Bar, 5 μm. (B to D) Fluorescence intensity profiles of representative cysts shown in (A) were generated to quantify the location of GRA protein(s) relative to the cyst wall, with DBA compared to α-GRA5 at day 7 for each method of permeabilization. The dotted black lines define the cyst wall region, and the dotted red line indicates the middle of the cyst wall, which corresponds to the peak DBA fluorescence intensity.

**FIG 5:**
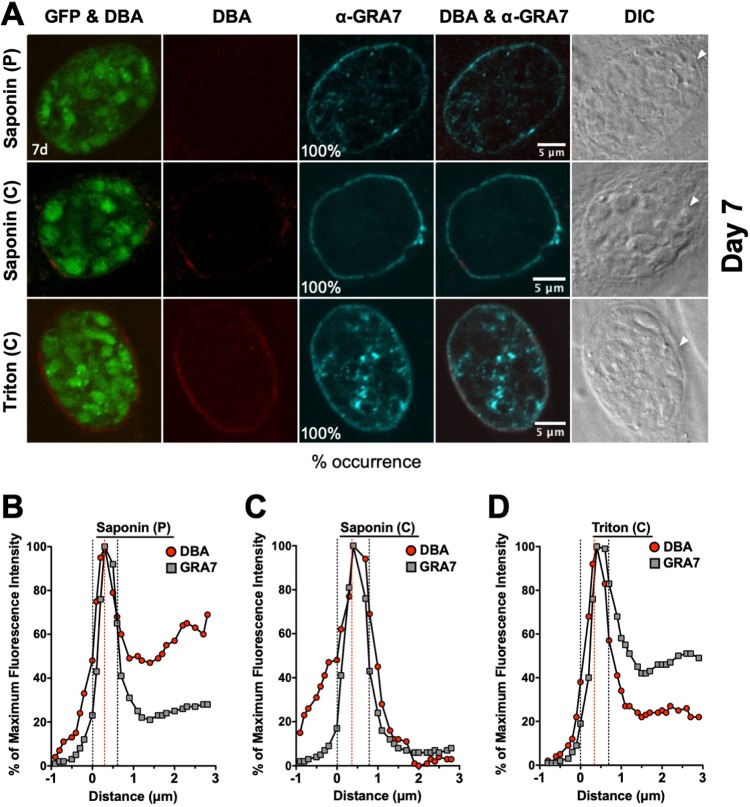
Localization of GRA7 in 7-day-old mature cysts. (A) Infected HFFs on coverslips were treated under bradyzoite-inducing conditions for 7 days to differentiate mature *in vitro* cysts. Cysts were located using DIC microscopy and imaged by confocal microscopy. The presence of bradyzoites inside cysts was verified by locating parasite nuclei with DAPI staining (not shown) and verifying that each parasite nucleus was surrounded by expression of cytosolic GFP (GFP^+^ bradyzoites). Cysts fixed in 4% paraformaldehyde were permeabilized with either a short pulse (P) of saponin, with continuous (C) exposure to saponin, or with continuous (C) exposure to Triton. Cysts were stained with DBA and α-GRA7 antibody. Panels show GFP and DBA, DBA, α-GRA7, DBA and α-GRA7, and DIC (cyst wall indicated by white arrow). The percent occurrence is shown for GRA7 at the cyst membrane/wall with saponin (P) (*n *= 16), saponin (C) (*n *= 16), and Triton (*n *= 15). Bar, 5 μm. (B to D) Fluorescence intensity profiles of representative cysts shown in (A) were generated to quantify the location of GRA protein(s) relative to the cyst wall, with DBA compared to α-GRA7 at day 7 for each method of permeabilization. The dotted black lines define the cyst wall region, and the dotted red line indicates the middle of the cyst wall, which corresponds to the peak DBA fluorescence intensity.

GFP^+^ bradyzoites were visible in DBA-stained cysts (Fig. 4 and 5). Peripheral DBA stain was detected at the cyst wall after permeabilization with a pulse of saponin. In contrast to 1- to 3-day-old cysts, the 7-day-old cyst wall was more intensely stained with DBA after permeabilization with Triton or continuous saponin treatment (Fig. 4 and Fig. 5), indicating that increased permeabilization of the 7-day-old cyst membrane provided greater access to the cyst wall.

GRA5 was exclusively observed at the cyst periphery after permeabilization with saponin pulse (Fig. 4A). After permeabilization with continuous saponin treatment, GRA5 was consistently observed at the cyst periphery and also as puncta in the cyst matrix. However, after permeabilization with Triton, GRA5 was not observed at the cyst periphery and was localized to puncta in the cyst matrix. In contrast, GRA7 was exclusively observed at the cyst periphery after permeabilization with saponin pulse or continuous saponin treatment, and GRA7 was found at the cyst periphery and in the cyst matrix after permeabilization with Triton (Fig. 5A). To quantitatively assess the location(s) of GRA5 and GRA7 in the mature 7-day-old cyst, we measured the cyst fluorescence intensity profiles for DBA and GRA5 or GRA7, respectively, using a previously reported Fiji macro (54). The cyst wall region in 7-day-old cysts occupied 6 layers (Fig. 4B to D and 5B to D). After permeabilization with a saponin pulse, the fluorescence intensity peak of GRA5 overlapped with the DBA fluorescence intensity peak and the maximum fluorescence intensity region of GRA5 was concentrated toward the left of the DBA fluorescence intensity region (Fig. 4B), indicating that GRA5 was localized toward the exterior of the DBA stain (cyst wall) and suggesting that GRA5 is a component of the cyst membrane (Fig. 4B). However, the fluorescence intensity peak of GRA5 overlapped with the DBA fluorescence intensity peak when cysts were permeabilized with continuous saponin treatment, showing that GRA5 was present throughout the cyst wall and cyst membrane region (Fig. 4C).

In contrast to saponin permeabilization, the GRA5 fluorescence intensity peak was shifted to the right of the DBA fluorescence intensity peak after permeabilization with Triton, suggesting that GRA5 was then localized to the inner layers of the cyst wall and in the cyst matrix (Fig. 4D). In contrast to GRA5, the fluorescence intensity peak of GRA7 overlapped with the fluorescence intensity peak of DBA, confirming that GRA7 was present throughout the cyst wall and cyst membrane region after any permeabilization condition (Fig. 5B to D). These results reveal that GRA5, unlike GRA7, predominantly interacts with the cyst membrane and cyst wall in a manner that is disrupted when cysts are permeabilized with Triton.

### Localization of GRA5 and GRA7 in mature 10-day-old cysts.

**FIG 6:**
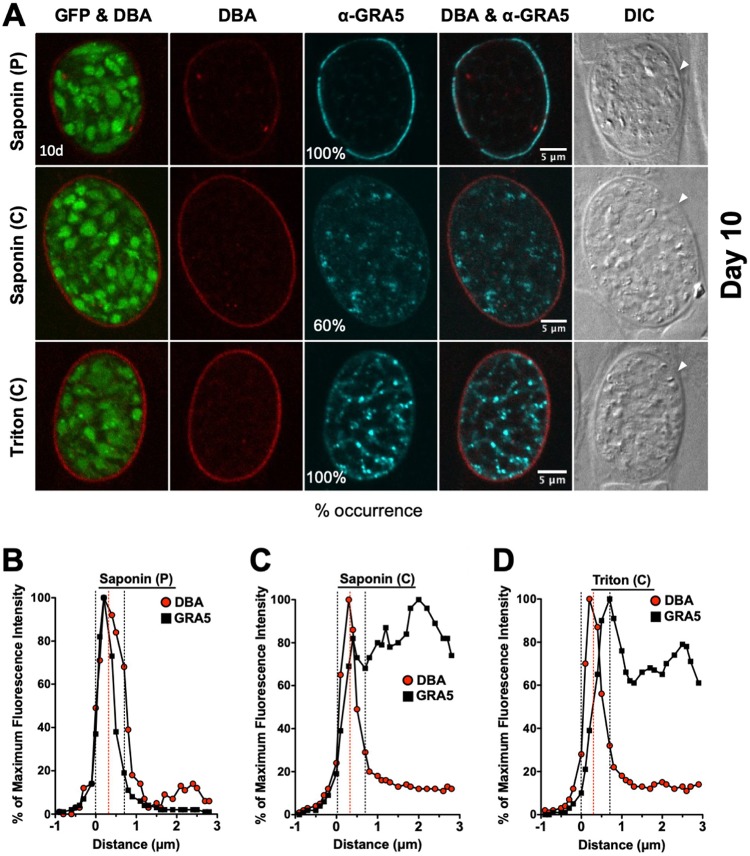
Localization of GRA5 in 10-day-old mature cysts. (A) Infected HFFs on coverslips were treated under bradyzoite-inducing conditions for 10 days to differentiate mature *in vitro* cysts. Cysts were located using DIC microscopy and imaged by confocal microscopy. The presence of bradyzoites inside cysts was verified by locating parasite nuclei with DAPI staining (not shown) and verifying that each parasite nucleus was surrounded by expression of cytosolic GFP (GFP^+^ bradyzoites). Cysts fixed in 4% paraformaldehyde were permeabilized with either a short pulse (P) of saponin, with continuous (C) exposure to saponin, or with continuous (C) exposure to Triton. Cysts were stained with DBA and α-GRA5 antibody. Panels show GFP and DBA, DBA, α-GRA5, DBA and α-GRA5, and DIC (cyst wall indicated by white arrow). The percent occurrence is shown for GRA5 with saponin (P) (*n *= 14; 14/14 GRA5 at the cyst membrane/wall), saponin (C) (*n *= 10; 6/10 GRA5 not at the cyst membrane/wall), and Triton (*n *= 11; 11/11 GRA5 not at the cyst membrane/wall). Bar, 5 μm. (B to D) Fluorescence intensity profiles of representative cysts shown in panel A were generated to quantify the location of GRA protein(s) relative to the cyst wall, with DBA compared to α-GRA5 at day 10 for each method of permeabilization. The dotted black lines define the cyst wall region, and the dotted red line indicates the middle of the cyst wall, which corresponds to the peak DBA fluorescence intensity.

**FIG 7:**
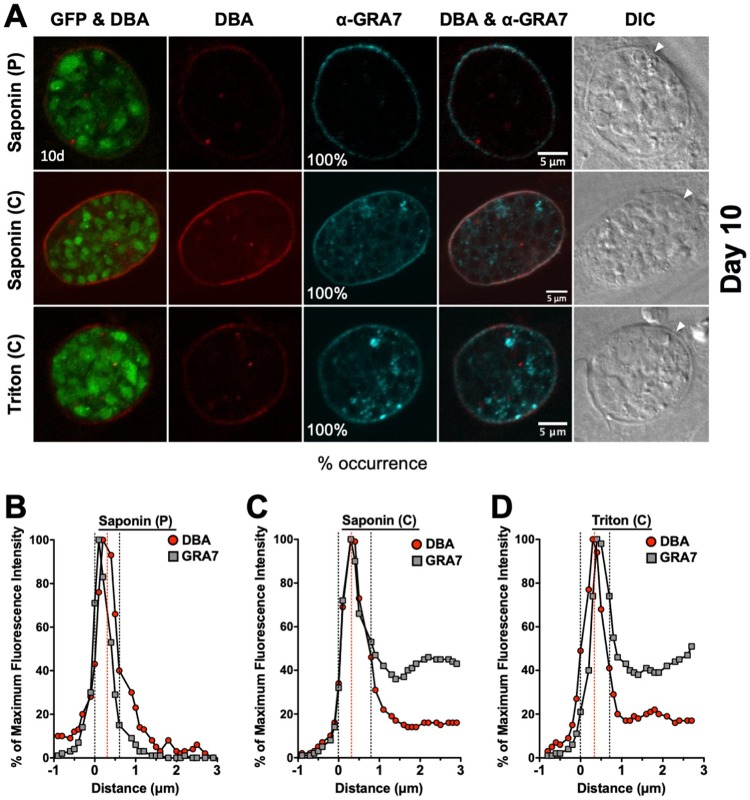
Localization of GRA7 in 10-day-old mature cysts. (A) Infected HFFs on coverslips were treated under bradyzoite-inducing conditions for 10 days to differentiate mature *in vitro* cysts. Cysts were located using DIC microscopy and imaged by confocal microscopy. The presence of bradyzoites inside cysts was verified by locating parasite nuclei with DAPI staining (not shown) and verifying that each parasite nucleus was surrounded by expression of cytosolic GFP (GFP^+^ bradyzoites). Cysts fixed in 4% paraformaldehyde were permeabilized with either a short pulse (P) of saponin, with continuous (C) exposure to saponin, or with continuous (C) exposure to Triton. Cysts were stained with DBA and α-GRA7 antibody. Panels show GFP and DBA, DBA, α-GRA7, DBA and α-GRA7, and DIC (cyst wall indicated by white arrow). The percent occurrence is shown for GRA7 at the cyst membrane/wall with saponin (P) (*n *= 21), saponin (C) (*n *= 16), and Triton (*n *= 12). Bar, 5 μm. (B to D) Fluorescence intensity profiles of representative cysts shown in panel A were generated to quantify the location of GRA protein(s) relative to the cyst wall, with DBA compared to α-GRA7 at day 10 for each method of permeabilization. The dotted black lines define the cyst wall region, and the dotted red line indicates the middle of the cyst wall, which corresponds to the peak DBA fluorescence intensity.

Similar to 7-day-old cysts, DBA stain was continuously detected at the cyst wall after permeabilization with a pulse of saponin. However, DBA staining of the cyst wall was more prominent after permeabilization with Triton or continuous saponin treatment (Fig. 6 and 7). After permeabilization with a saponin pulse, GRA5 was exclusively observed at the cyst periphery (Fig. 6A), and the fluorescence intensity peak of GRA5 overlapped with the fluorescence intensity peak of DBA, indicating the presence of GRA5 in the cyst wall and cyst membrane (Fig. 6B). Compared to GRA5, GRA7 exhibited an essentially identical pattern of localization to the cyst wall and cyst membrane after permeabilization with a saponin pulse (Fig. 7A). The GRA7 fluorescence intensity peak was slightly shifted to the left of the DBA fluorescence intensity peak, suggesting that GRA7 was primarily localized in the cyst membrane and the outer layer of the cyst wall (Fig. 7B).

After permeabilization of 10-day-old cysts with continuous saponin treatment, different localization patterns of GRA5 and GRA7 were observed, which revealed differences in how GRA5 and GRA7 interacted with the cyst membrane and the cyst wall. While GRA7 was occasionally localized to puncta in the cyst matrix (Fig. 7A), the GRA7 and DBA fluorescence intensity peaks closely overlapped, revealing that GRA7 was localized in the cyst membrane and throughout the cyst wall region (Fig. 7C). In contrast, peripheral GRA5 stain was markedly reduced, and puncta of GRA5 were prominent in the cyst matrix, revealing an increased susceptibility to continuous saponin treatment (Fig. 6A). Correspondingly, GRA5 fluorescence intensity peaks were shifted to the right of the DBA fluorescence intensity peak, revealing that GRA5 was absent from the cyst membrane and was present in the inner layers of the cyst wall and in the cyst matrix (Fig. 6C).

After permeabilization of 10-day-old cysts in Triton, GRA5 and GRA7 exhibited similar patterns of localization. Peripheral GRA5 (Fig. 6A) and GRA7 (Fig. 7A) staining was reduced and prominent GRA5 and GRA7 puncta were found in the cyst matrix. Correspondingly, the GRA5 (Fig. 6D) and GRA7 (Fig. 7D) fluorescence intensity peaks were shifted to the right of the DBA fluorescence intensity peak, suggesting that GRA5 and GRA7 were absent from the cyst membrane and were localized to the inner layers of the cyst wall and in the cyst matrix.

## DISCUSSION

The bradyzoite-stage cyst membrane and cyst wall structures form a barrier that maintains a chronic infection (69) and poses a therapeutic obstacle for cyst elimination (70). Our study tracked the location(s) of PVM-associated GRA5 and GRA7 from differentiation of the PV to the mature 10-day-old *in vitro* cyst. Our observations confirm a recent proteomic study of the cyst wall that identified GRA5 and GRA7 in the cyst wall of *in vitro* cysts matured for 8 days (53). Furthermore, GRA5 has been previously observed at the cyst membrane in mature cysts (63, 66). In contrast, GRA7 was previously localized to membrane tubule components of the intracyst network (ICN) in the cyst matrix, a structure that resembles the IVN, as well as to convoluted membranes that were present in the cyst wall (56). Our results show that after permeabilization using a saponin pulse, GRA5 and GRA7 were localized to the cyst membrane as well as to the cyst wall region. In addition, by tracking the localization of GRA5 and GRA7, our results suggest that GRA5 and GRA7 are consistently localized to the cyst membrane and cyst wall region at all times after differentiation of the tachyzoite stage PV, supporting a previous model that proposed that the PVM develops into the cyst membrane (56).

Measurement of DBA stain localization in Δ*gra3*, Δ*gra5*, Δ*gra7*, Δ*gra8*, and Δ*gra14 in vitro* cysts revealed that PVM GRAs were crucial to support the normal rate of accumulation of cyst wall proteins at the cyst periphery. These results mirror recent results we reported for IVN Δ*gra12* (38), and Δ*gra2*, Δ*gra4*, Δ*gra6*, and Δ*gra9 in vitro* cysts (54) and suggest that both IVN- and PVM-localized GRAs are crucial for the development and the maturation of the cyst wall and cyst membrane. These findings support the hypothesis that membranes at the cyst periphery are occupied by GRA proteins that play key roles in the development and maturation of the cyst wall and cyst membrane. The functions of some of the PVM GRAs have been reported in the tachyzoite-stage PV. GRA3 is a type I transmembrane protein that localizes to the PVM and IVN (71), and GRA3 molecules were observed at the cyst wall (56). GRA3 interacts with calcium-modulating ligand (CAMLG), a type II transmembrane protein of ER (72), and it recruits and engulfs host Golgi into the PV (73). GRA5 is a type I transmembrane protein that is targeted to the PVM (64). GRA5 molecules are concentrated at the cyst periphery (67) and associate with the cyst membrane (63, 66). GRA7 is a transmembrane protein that localizes to the PVM, IVN (74), and membranous PV extensions (PVE) that extend from the PVM into the host cytosol (68). GRA7 molecules associate with ROP2 and ROP4 (75) and interact with ROP5/ROP18 complex at the PVM to regulate the ROP18-specific inactivation of Irga6 to resist IFN-γ-activated host cell innate immunity (76). GRA7 acts as a garroting protein that sequesters host endolysosomes within the tachyzoite-stage PV (77). GRA7 tubulates artificial liposomes, and targeted deletion of GRA7 under serum-deprived conditions leads to slow tachyzoite-stage parasite growth (77). GRA7 molecules were observed in association with membranes in the cyst wall and the cyst matrix (56). GRA8 is a proline-rich protein that localizes to the PVM (78) and is a component of the subpellicular cytoskeleton in tachyzoite-stage parasites (79). GRA14 is a transmembrane protein that localizes to the PVM, IVN, and PVE (80). GRA14 can be transferred between PVs via PVEs (80). Deletion of GRA3 (81), GRA7, GRA8, and GRA14 did not affect the parasite’s ability to differentiate *in vitro* but markedly reduced cyst burden *in vivo* (38). However, the specific role(s) of these PVM GRA molecules in the cyst wall and cyst membrane during cyst development still remains to be determined.

Intriguingly, depletion of low-density lipoprotein (LDL)-derived cholesterol increases tachyzoite-to-bradyzoite stage conversion (82). Our findings show that the cyst membrane of immature cysts was differentially permeabilized by saponin and Triton X-100 (Triton) detergent treatments. Saponin selectively interacts with cholesterol in the lipid bilayer membrane, causing the formation of permeability holes (83). In contrast, Triton is a nonionic detergent that inserts into membranes to dissolve lipid-lipid and lipid-protein interactions, while typically preserving protein-protein interactions (84). In immature cysts 3 days old or younger, DBA staining of the cyst wall was more intense after permeabilization in Triton in comparison to saponin pulse. However, this difference was not observed in mature 7- or 10-day-old cysts, and DBA staining of the cyst wall was equivalent with both detergents. In addition, DBA staining intensity varied at different times of cyst development after permeabilization using a saponin pulse. DBA staining of 6-h-old cysts was apparent. In contrast, DBA staining of immature 1-, 2-, and 3-day-old cysts was not detected. However, in 7- and 10-day-old mature cysts, the cyst wall was DBA stained after saponin pulse permeabilization. These DBA staining patterns of the cyst wall after saponin pulse permeabilization suggest that the lipid composition of the cyst membrane, and perhaps those of other membranes present in the cyst wall, such as the ICN membranes (56), varies during cyst development. Detection of DBA staining in 6-h-old cysts but not in 1-, 2-, or 3-day-old cysts suggests that the cholesterol content of the cyst membrane may decrease between 6- and 24-h postdifferentiation. If so, this would mirror the fate of cholesterol in the PVM after tachyzoite invasion, which is highest in the newly formed PVM and is markedly reduced in the old PVM after several rounds of tachyzoite replication have occurred (85). The higher cholesterol content of the early PVM appears to arise via an invasion mechanism involving the formation of cholesterol-rich evacuoles that fuse to the nascent PVM (85–87). In addition, after immature cysts developed into mature 7- and 10-day-old cysts, the cyst membranes became more saponin sensitive and exhibited increased DBA staining of the cyst wall after saponin pulse permeabilization. The increased saponin sensitivity of the cyst membranes in mature cysts is consistent with the ability of 3- and 7-day-old *in vitro* cysts to contain more cholesterol-containing lipid droplets derived from the host cell (88). Thus, while it is tempting to speculate that the cholesterol content of the cyst membrane increases as cyst mature, other interpretations of these results are possible, and future experiments are necessary to measure the cholesterol content in cyst membranes during cyst development.

The glycosylated cyst wall forms beneath the cyst membrane. How cyst wall cargo is delivered to the cyst periphery remains unknown. A favored hypothesis is that wall cargo is delivered in vesicles secreted by bradyzoite-stage parasites. Our data revealed bright puncta of GRA5 and GRA7 close to the cyst periphery at 6 h after differentiation. This phenotype is similar to previous findings that GRA1, GRA2, GRA4, GRA6, GRA9, and GRA12 were observed in puncta at the cyst periphery 6 h after differentiation (54). It is tempting to speculate that these puncta represent secreted vesicles; however, future electron or high-resolution microscopy studies are needed to investigate the mechanisms that direct the delivery of cyst wall cargo to the developing cyst wall.

To assess the proximity of PVM GRAs, we used a previously developed quantitative method (54), which defined the cyst wall region to allow the fluorescence intensity of GRA5 and GRA7 in relation to the DBA-stained cyst wall to be measured.

The initial PVM is hypothesized to undergo changes that culminates in a limiting cyst membrane. Cyst wall proteins congregate at the cyst periphery (54), presumably underneath the cyst membrane, to develop the cyst wall. The mature cyst membrane has been observed to be noncontinuous (ruffled) and is present in the outer layers of the cyst wall (49, 53, 56). The cyst wall structure has two recognizable layers, a densely compacted outer layer and a less densely compacted inner layer that faces the cyst matrix (56). Using these cyst membrane and cyst wall descriptions in conjunction with our cyst wall analysis, we localized GRA5 and GRA7 in the cyst membrane since their fluorescence intensity peaks were observed outside and exterior to the DBA-stained cyst wall fluorescence intensity peak. In addition, GRA5 and GRA7 were localized throughout the cyst wall, suggesting that GRA5 and GRA7 also associate with the ICN membranes that penetrate into the cyst wall from the cyst matrix (56).

When mature 7- or 10-day-old cysts were permeabilized with Triton, GRA5 and GRA7 were localized in the inner layers of the cyst wall and in the cyst matrix as prominent puncta. Remarkably, saponin permeabilization caused differential localization of GRA5 and GRA7 selectively in 10-day-old mature cysts. GRA5 was localized to the inner layers of the cyst wall and to prominent puncta in the cyst matrix after continuous saponin permeabilization. In contrast, GRA7 was localized at the cyst membrane and cyst wall region. The simplest explanation for this differential localization is that GRA5 most likely resides in cholesterol-rich lipid rafts in the cyst membrane, whereas GRA7 does not. Alternatively, GRA7 may interact with a protein component of the cyst wall that stabilizes its cyst membrane localization after saponin permeabilization, but, as mentioned earlier, not after Triton permeabilization. Elucidating the functions of PVM GRAs during cyst development is necessary to define the mechanisms that regulate the development and maturation of the cyst wall and cyst membrane.

## MATERIALS AND METHODS

### Culture conditions and strains.

Type II Prugniaud (Pru) background Toxoplasma gondii parasites were maintained *in vitro* by serial passage of tachyzoites in human foreskin fibroblast (HFF) monolayers (ATCC SCRS-1041.1) cultured in Eagle’s modified essential medium (EMEM; Lonza) containing 1% fetal bovine serum (FBS; Life Technologies), 2 mM glutamine, 100 units/ml penicillin, and 100 μg/ml streptomycin at 36°C in 95% air and 5% CO_2_. HFF cells were maintained in EMEM, 10% FBS (HyClone), 2 mM glutamine, 100 units/ml penicillin, and 100 μg/ml streptomycin at 37°C in 95% air and 5% CO_2_. The parental Pru strain PruΔ*ku80* was previously made transgenic for green fluorescent protein (GFP) under the control of the LDH2 bradyzoite stage-specific promoter (89). Strains used in this study were developed using the Δ*ku80* knockout strain of the type II Pru strain using previously described methods (38, 90).

### *In vitro* cyst differentiation assay.

Tachyzoites were differentiated *in vitro* into bradyzoites within cysts essentially as previously and elegantly described by Knoll and colleagues (57). Differentiation medium contained Roswell Park Memorial Institute medium (RPMI) without bicarbonate supplemented with 2.05 mM l-glutamine (HyClone), 20 mM HEPES-free acid (IBI Scientific), 1% XL-glutamine (a long-lasting stable form of glutamine; VWR), 1% FBS, and 1% penicillin-streptomycin. The pH of differentiation medium was adjusted to 8.1 with sodium hydroxide and filter sterilized. HFF cells were cultured on circular micro cover glass until confluent (Electron Microscopy Sciences), and confluent monolayers were infected with type II Pru parasites at a multiplicity of infection (MOI) of ∼0.5. Infected cells were washed 3 h after infection once in Dulbecco’s phosphate-buffered saline (DPBS) supplemented with Ca^2+^ and Mg^2+^ and incubated in differentiation medium for 6 h, 1 day, 2 days, 3 days, 7 days, or 10 days at 37°C in ambient air. Medium was changed on days 3 and 7.

### Cyst immunofluorescence assay and cyst locating.

Infected cells were fixed in 4% paraformaldehyde for 10 min, and the excess was quenched with 0.1 M glycine. Infected cells were permeabilized and blocked in either (i) 0.01% saponin (Sigma) for 10 min, (ii) 3%FBS/0.01% saponin for 30 min at room temperature (RT), with this permeabilization solution used throughout the experiment, or (iii) 3%FBS/0.2% Triton X-100 for 30 min at RT, with this permeabilization solution used throughout the experiment. All samples were incubated with a 1:1,000 dilution of primary mouse monoclonal α-GRA5 antibody (63) or with a 1:1,000 dilution of primary rabbit α-GRA7 (91) (antibodies purchased from Biotem [Apprieu, France] or kindly provided by D. Jacobs, Innogenetics-Fujire-bio Europe N.V. [Ghent, Belgium]). Preparations were washed three times with DPBS supplemented with Ca^2+^ and Mg^2+^ and incubated for 1 h at RT with a 1:1,000 dilution of secondary goat anti-rabbit (H+L) (Thermo Fisher) and goat anti-mouse IgG (H+L) antibodies conjugated to Alexa Fluor 647 (Cell Signaling). All samples were incubated with a 1:250 dilution of rhodamine-labeled Dolichos biflorus agglutinin (Vector Laboratories) for 1 h at RT. The Pru background used in this study has a bradyzoite-specific gene, LDH2, under a GFP promoter (89), which is expressed when the parasites switch from tachyzoites to bradyzoites. Samples were mounted in SlowFade Gold antifade with 4′,6-diamidino-2-phenylindole (DAPI; Life Technologies) and then imaged with a Nikon A1Rsi confocal microscope (Nikon, Inc.) using an Apo TIRF 100× oil differential interference contrast (DIC) N20 objective. Cysts were randomly selected for analysis by locating cysts using DIC microscopy. Bradyzoite differentiation in cysts was confirmed by GFP^+^ bradyzoites. The focal plane (from a z-stack) selected for quantification was from the middle of the cyst, where the cyst size is maximal. Raw .nd2 files of cyst images were imported into Fiji for processing. Images were minimally processed for brightness (image → adjust → color balance) in Fiji (92). The number of cysts for each strain analyzed in each experiment is shown in the figure legends for Fig. 1 to 7 and Supplemental Fig. S1 and S2. The percent occurrence was calculated as the number of cysts showing the representative phenotype that are shown in the representative image out of the total number of cysts imaged.

### Cyst fluorescence intensity profiles.

Raw .nd2 image files were imported into Fiji to measure fluorescence intensity parallel to the cyst wall as previously described (54). Images were cropped to isolate each cyst. A macro was written to generate a reliable mask of the cyst, slightly outside the cyst wall, using the DBA-rhodamine channel. The DBA-rhodamine channel was used to threshold the cyst, and holes were filled inside to get a continuous mask of the whole cyst. Successive layers were generated based on the original mask, growing or shrinking using the “dilate” or “erode” morphological operations. Layers were generally 1 pixel thick. The fluorescence intensity of each region was measured for a selected fluorescent channel, DBA, GRA5, or GRA7. The macro generated layers within the cyst until the minimum area of the (shrinking) layer reached 1,000 pixels^2^. Layers were created by dilation to measure the fluorescence intensity outside the cyst, which provided the background fluorescence intensity. All data were imported into Excel to be further analyzed, as previously described (54). Calculated percentage of maximum fluorescence intensity and distance (μm) values were imported and graphed in Prism.

### Cyst wall definition and analysis.

The cyst wall region was identified and defined as previously described (54). The cyst wall outer region was identified by DBA, while the inner region was determined by GFP, which identifies the parasites within the cyst. The cyst wall region is defined by outer and inner boundaries, which were determined by the first point less than 50% of maximum fluorescence intensity of DBA and GFP, respectively. The cyst wall region is marked by dotted black lines, and the peak of DBA fluorescence is marked by a dotted red line. Next, we evaluated location of GRA5 or GRA7 in comparison to the DBA-stained cyst wall using fluorescence intensity measured at the same time within the cyst. This cyst wall analysis was used to determine if two proteins were observed in the same layer.

### Cyst total fluorescence intensity quantification assay.

Raw .nd2 image files were imported into Fiji to measure total fluorescence intensity at the cyst periphery and within the cyst interior, as previously described (54). The cyst periphery was determined to be the cyst wall plus two layers, which were added to include proteins near the cyst wall but not yet incorporated into the cyst wall. Fluorescence for DBA was measured in Δ*ku80*, Δ*gra3*, Δ*gra5*, Δ*gra7*, Δ*gra8*, and Δ*gra14* strains. To measure background fluorescence, a circle was drawn using the freehand selection tool, and fluorescence was measured outside the cyst on three different sides. All data were imported into Excel to be further analyzed as previously described (54). All ratios were entered and graphed in Prism. A ratio of <1 means there is greater DBA fluorescence intensity in the cyst interior compared to that in the cyst periphery, a ratio of 1 represents an equal DBA fluorescence intensity at the cyst periphery to that in the cyst interior, and a ratio of >1 means there is greater DBA fluorescence intensity at the cyst periphery than in the cyst interior. *P* values were calculated with a Student’s *t* test; ****, *P* < 0.0001.

### Immunofluorescence assay for tachyzoites.

HFFs were cultured on circular micro cover glass and were infected with parasites for 24 h. Samples were fixed in 4% paraformaldehyde for 10 min, permeabilized with 0.01% saponin (Sigma) for 10 min, and blocked with 10% FBS for 20 min. All samples were incubated with a 1:250 dilution of rhodamine-labeled Dolichos biflorus agglutinin (Vector Laboratories) for 1 h at RT. All samples were mounted in SlowFade Gold antifade with DAPI (Life Technologies) and imaged at ×100 with a Nikon A1Rsi confocal microscope (Nikon, Inc.). Vacuoles were located using differential interference contrast (DIC) microscopy. Confocal images as raw .nd2 files were imported and minimally processed for brightness in Fiji (92).

### Statistical analysis.

Unpaired *t* tests were used to calculate *P* values. All calculations of average, plus or minus standard error of the mean (SEM), and *P* values were performed using GraphPad Prism software version 5.0c.
